# Higher-order Multivariable Polynomial Regression to Estimate Human Affective States

**DOI:** 10.1038/srep23384

**Published:** 2016-03-21

**Authors:** Jie Wei, Tong Chen, Guangyuan Liu, Jiemin Yang

**Affiliations:** 1 School of Electronic and Information Engineering, Southwest University, Chongqing, 400715, China; 2 Chongqing Key Laboratory of Non-linear Circuit and Intelligent Information Processing, Southwest University, Chongqing, 400715, China; 3 Faculty of Psychology, Southwest University, Chongqing, 400715, China

## Abstract

From direct observations, facial, vocal, gestural, physiological, and central nervous signals, estimating human affective states through computational models such as multivariate linear-regression analysis, support vector regression, and artificial neural network, have been proposed in the past decade. In these models, linear models are generally lack of precision because of ignoring intrinsic nonlinearities of complex psychophysiological processes; and nonlinear models commonly adopt complicated algorithms. To improve accuracy and simplify model, we introduce a new computational modeling method named as higher-order multivariable polynomial regression to estimate human affective states. The study employs standardized pictures in the International Affective Picture System to induce thirty subjects’ affective states, and obtains pure affective patterns of skin conductance as input variables to the higher-order multivariable polynomial model for predicting affective valence and arousal. Experimental results show that our method is able to obtain efficient correlation coefficients of 0.98 and 0.96 for estimation of affective valence and arousal, respectively. Moreover, the method may provide certain indirect evidences that valence and arousal have their brain’s motivational circuit origins. Thus, the proposed method can serve as a novel one for efficiently estimating human affective states.

In order to establish affective human-computer interactions, improve diagnostic-therapy effects for mental disorders, and reveal psychophysiological mechanisms of emotion, it is a crucial first step to detect human affective states from direct observations, neural activations, facial videos, voice recordings, body gestures, and physiological signals, etc.^1^,^2^,^3^,^4^,^5^,^6^,^7^,^8^,^9^. In the area of human-computer interaction (HCI), automatically recognizing and responding to a user’s affective states during interactions with a computer can enhance the quality of the interaction to maximize user’s pleasure^2^,^3^,^4^,^10^. In mental care, detecting human affects by technological systems and devices has quantitative, objective, and automatic accessory diagnostic value for simple mood questionnaires or interviews that are commonly used to diagnose pathological emotional fluctuations^6^,^7^,^9^. Further more, the methods, models, and conclusions, that are developed in the field of Affective Computing (AC) and used to senor human affects, can supply certain valuable references for other scientists to reveal and prove the theoretical and neural psychophysiological mechanisms of human affect to enhance human mental health^3^,^7^,^11^.

The affective detection (AD) takes feature vectors extracted from observed signals as input and assigns psychological affective labels to these feature vectors. In current AD researches, there are two main directions: (I) classification, in which the state of art machine learning algorithms are applied to classify and recognize different affective states (e.g., ‘happy’, ‘anger’, ‘sad’, and ‘fear’, etc.) and (II) estimation, in which function approximation methods are adopted to approximate the complex mapping relationships between observations and their corresponding psychological states, and then affective states (e.g., the continuous scores of valence and arousal) are computed by obtained function models (see Fig. 1). Since Rosalind Picard published the most influential works^1^,^2^, most of researchers have been enthusiastic about obtaining high recognition accuracy by inventing and improving machine learning algorithms, feature extracting methods, signal processing methods, and affective induction experiments^12^,^13^,^14^,^15^,^16^,^17^,^18^,^19^,^20^. Intensive reviews about classifying affective states can be found in existing works^3^,^18^,^21^,^22^,^23^,^24^,^25^,^26^,^27^. Compared with classification methods, estimation methods have been used far less frequently^5^,^28^ partly because of lacking knowledges of complex psychophysiological mechanisms of human affect. In fact, classification and estimation methods are complementary to each other. Their external main differences are that classification methods label psychological states in a discrete categorical manner, while estimation methods regard psychological states as a continuous process. Estimating individual affective states in a continuous score manner is one important aspect of AD because it is more common for people to exhibit their affective experiences in a continuous manner during everyday communications^25^,^29^. Further more, continuously estimating individual affect can supply more quantitative results than what classification procedures can supply. Methods of estimating human affective states, such as multivariate linear-regression analysis (MLR), partial least-square estimation (PLS), genetic algorithm optimized support vector regression (GA-SVR), artificial neural network (ANN), bidirectional long short-term memory neural networks (LSTM-NNs), fuzzy logical analysis (FLA), and sequence Bayessian analysis (SBA), have been proposed in the past decade^28^,^29^,^30^,^31^,^32^,^33^,^34^. Although their performances differ with each other, these computational models achieve relative good results to some extent, and supply us a good prospect in affective estimation. It has been well-accepted by the scientific community that affective psychophysiological processes should be complex nonlinear ones. In general, the MLR models are lack of precision because of ignoring intrinsic nonlinearities of complex psychophysiological processes. The PLS and SVR models have their limitations in processing nonlinearities by adopting finite kernel functions. The rest models commonly adopt depth mathematics and complicated algorithms that are not easily understood and convenient to practically implement affective estimation. Moreover, they commonly regard affective psychophysiological processes as black boxes to be modeled and are lack of actual psychophysiological bases.

To improve estimation accuracy and simplify model, the study specifically introduces the higher-order multivariable polynomial regression (HMPR) method to approximate the implicit complex nonlinear function relationships between observed response patterns and corresponding psychological states, based on the Taylor theorem in mathematical analysis that any smooth function can be approximated by its Taylor polynomial (a higher-order multivariable polynomial) at any precision in the convergent domain. The interactions among diverse physiological systems^35^,^36^ and overlapped central nervous system structures of the affective motivational brain system and skin conductance^9^,^37^ are the psychophysiological foundations of modeling the skin conductance response (SCR) affective process that translates pure SCR patterns into their corresponding affective states (model hypothesis in Methods). Moreover, multivariable regression analysis theory and methods were applied to model many other processes very well (e.g., chemical systems, scattered data sets in computational physics, the volume computation problem of forest trees, and major depressive disorder problems, etc.)^38^,^39^,^40^,^41^,^42^,^43^. In order to solve the key technical problem of requiring large data sets in executing the HMPR, we propose a statistical formula (equation (4) in Methods) to construct the simulated data sets, which is larger than the experimental data sets. We carried out the HMPR on the simulated data sets to obtain the optimal higher-order multivariable polynomial model (HMPM), and tested the obtained final HMPM on the entire experimental data sets to obtain its accuracy. A general overview of the proposed estimation method and analysis are shown in Fig. 2. The results indicate that a simple HMPM is able to efficiently estimate the affective valence and arousal from pure skin conductance responses. This may be useful to develop affective smart wearable devices (e.g., Apple Watch, Mi Band, and Google Glass, etc.). Further more, through analyzing the gradient fields of the obtained HMPM, we offered certain indirect evidences to support that the valence factor is related with the activation of appetitive and defensive subcircuits, and the arousal factor may only reflect activation intensity^9^.

## Results

This section consists of data sets, the affective HMPM, and the comparison with the ANN method. The data sets contain the fundamental data sets, experimental data sets, and simulated data sets. Based on the simulated data sets, we adopted the HMPR to model the SCR affective process and obtained the optimal affective HMPM. The optimal affective HMPM was tested on the entire experimental data sets to obtain its final accuracy. Moreover, the comparison of the HMPR and commonly used ANN is given. More specific details are stated in the methods section. All of the analysis were implemented by using Matlab R2013b endowed with self-made codes and an additional Neural Network Toolbox for the ANN model.

### Data sets

**Figure 3:**
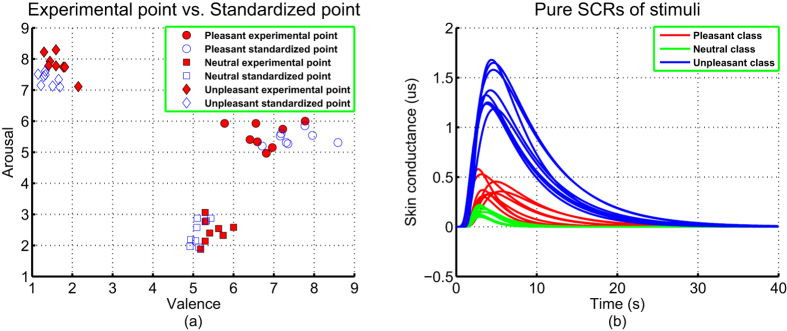
Affective and physiological patterns of experimental stimuli. (**a**) the experimental affective ratings and standard ratings are expressed into points in the affective valence-arousal space. For the experimental stimuli (24 pictures), standardized ratings in the IAPS are represented by hollow markers; and experimental ratings are represented by solid markers. (**b**) the physiological SCR patterns are expressed into their corresponding waveforms by using equation (3) (Methods) and parameters values in the Supplementary Table S1.

The fundamental data sets contain the affective pattern and physiological SCR pattern (see Supplementary Table S1). The valence-arousal ratings and affective physiological signals (Pulse, SC, and ECG) of each picture were obtained by the experimental protocol. For each picture, its affective pattern contains its mean valence and arousal scores across subjects. The good match between experimental and standardized affective ratings^44^ is illustrated in Fig. 3(a). The good distinctions among pleasant, neutral, and unpleasant affective ratings also show that three affective states were successfully induced in subjects in our experiment. For each picture, its physiological SCR pattern is a four dimensional feature vector that consists of onset time, gain, rise time, and decay time constant (Time constant) parameters. Applying the Lim’s nonlinear curve fitting^45^ with 20 step iterations, the four parameters are extracted from the SC signal which is obtained by averaging 10-second SC segments after picture presentation across subjects. It is seen from Fig. 3(b) that the pure SCR waveforms are obviously clustered into three classes. This finding is consistent with the view (supported by a considerable experimental literature) that both animals and humans show specific, autonomic reflex patterns in reaction to affective cues^9^,^24^.

**Figure 4:**
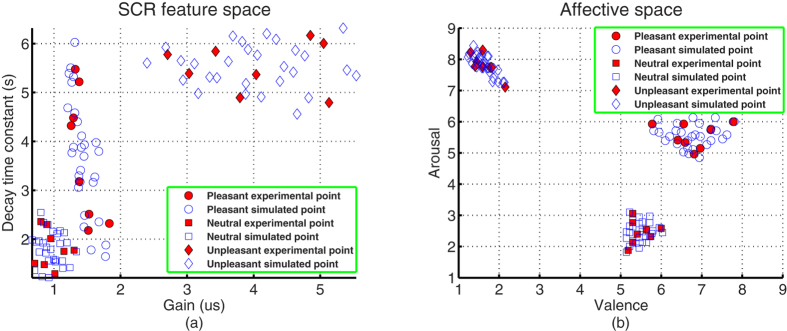
Experimental data vs. Simulated data. The experimental data sets are represented by solid markers, and the simulated data sets are represented by hollow markers. (**a**) Two dimensional SCR feature space. (**b**) Affective valence-arousal space.

The experimental data sets consists of gain, time constant, valence, and arousal columns of the fundamental data sets (see Supplementary Table S1). Applying the one-way ANOVA analysis on each column of the physiological SCR pattern, we find that there are statistical significant differences in three columns (onset time, gain, and time constant), and there is no statistical significant difference in one column (rise time) because the corresponding F-values are *F*(2, 21) = 9.55, 68.21, 2.12 and 38.25, and P-values are *P* = 0.0011, 6.2504*e* − 10, 0.1423 and 9.9679*e* − 8. Since rise time (no significant difference) can be regarded as a constant for the computing purpose^46^, the three dimensions (onset time, gain, and time constant) were regarded as the potential input variables of the SCR affective process. During the latter process of establishing the affective HMPM, we find that gain and time constant dimensions have significant contributions for estimating affective valence and arousal. Hence, the data of gain, time constant, valence, and arousal columns forms the experimental data sets of the study (see solid markers in Fig. 4).

The simulated data sets are constructed from the experimental data sets. For each affect and column of the experimental data sets, we arbitrarily chose two values to simulate a new value by using equation (4). All simulated new values form the simulated data sets (see Supplementary Table S2 and hollow markers in Fig. 4). It is obvious that the simulated data sets keep the patterns of the experimental data sets and contain more data points than those of the experimental data sets.

### Affective HMPM

**Figure 5:**
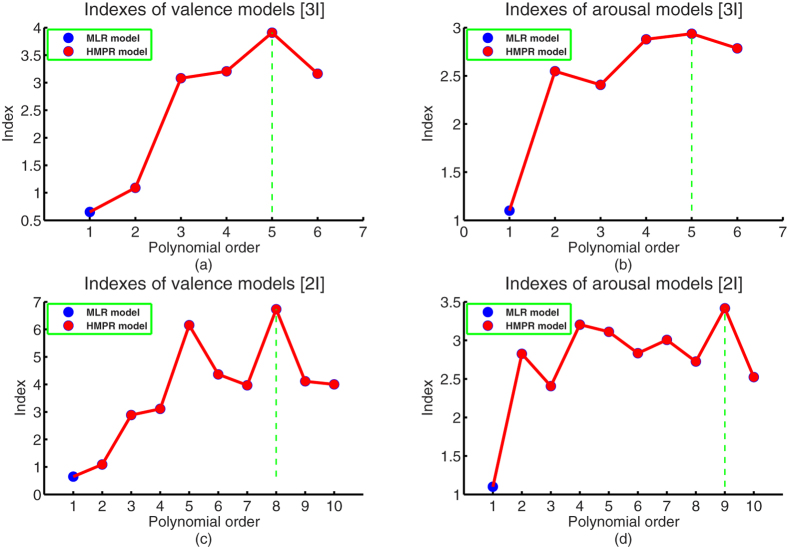
Indexes of HMPMs and MLR models. (**a**) six valence models’ Indexes, these valence models have three input variables, which are onset time, gain, and time constant. (**b**) six arousal models’ Indexes, these arousal models have the same input variables as models in (**a**). (**c**) ten valence models’ Indexes, these valence models have two input variables, which are gain and time constant. (**d**) ten arousal models’ Indexes, these arousal models have the same input variables as models in (**c**).

To find the best HMPM to estimate human affect from the physiological SCR pattern, we carried out the HMPR for valence and arousal factors, respectively, under the matrix singular criterion 10^−8^ and significant level 10^−4^. As previously mentioned, the onset time, gain, and time constant dimensions were regarded as the potential input variables of the SCR affective process. Based on the simulated data sets and the simulated values of the onset time dimension, we established five HMPMs and one MLR model for valence and arousal factors, respectively (a first order HMPM is a MLR model). Their performances are seen in the Supplementary Table S3. Their Indexes are presented in the upper panel of Fig. 5(a,b). The Index of a model is the ratio of its mean squared error (MES) to its Pearson’s correlation coefficient r (equation (8) in Methods). In general, the more accurate the model is, the bigger the correlation coefficient is, the smaller the mean squared error is, and hence the bigger the Index is. From the upper panel of Fig. 5, it is easy to find that HMPMs are generally better than MLR models, and that the fifth order HMPMs are the best for estimating valence and arousal from onset time, gain, and time constant dimensions (model see Supplementary Equation S1). By observing these two higher-order multivariable polynomial functions, we find that the onset time dimension has no significant effects on computing affective valence and arousal. Hence, we carry out the HMPR only on the simulated data sets with the same settings of the previous one again, and obtain nine HMPMs and one MLR model (with two inputs, gain and time constant) for valence and arousal factors, respectively. Their Indexes are seen in the Supplementary Table S4. Their Indexes are presented in the lower panel of Fig. 5(c,d). From the lower panel of Fig. 5, it is easy to find that HMPMs are again generally better than MLR models, and that the eighth order HMPM is the best for estimating valence and the ninth order HMPM is the best for estimating arousal from gain, and time constant dimensions. Hence, the final optimal HMPM with two input variables under significant level 0.0001 is as follows:


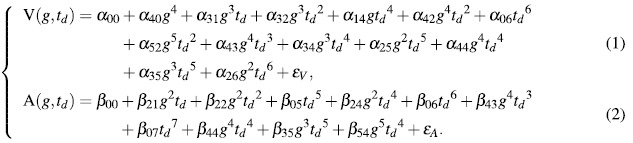


where *g* and *t*_*d*_ are gain and time constant, respectively, *α*_*ij*_ and *β*_*lk*_ are model parameters (see Supplementary Table S5), and the terms *ε*_*V*_, *ε*_*A*_ are errors. In fact, the affective HMPM (equations (1) and (2)) consists of two polynomial functions concerning input variables *g* and *t*_*d*_. Tested on the entire experimental data sets (solid markers in Fig. 4), the final performance of the affective HMPM is that r = 0.9801(0.9536, 0.9915), p-value = 6.1657*e* − 17, MSE = 0.1948, and Index = 5.0301 for valence estimation; and r = 0.9600(0.9084, 0.9828), p-value = 1.1940*e* − 13, MSE = 0.3894, and Index = 2.4654 for arousal estimation.

**Figure 6:**
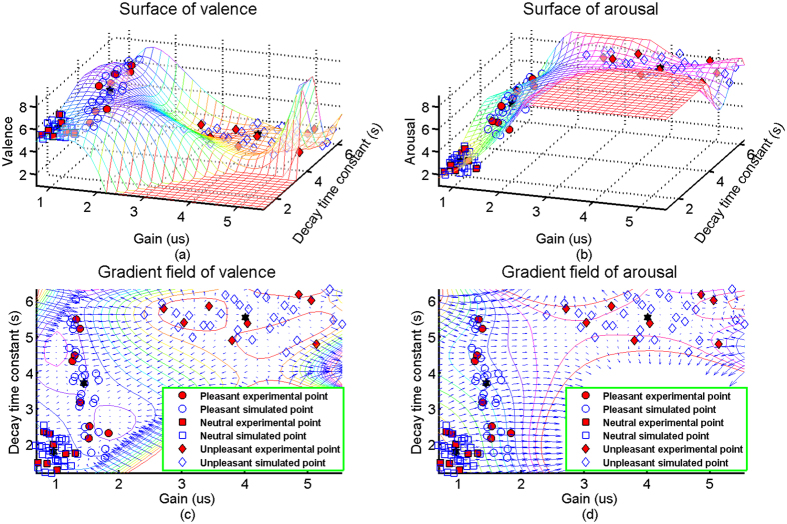
Surfaces and gradient fields of the affective HMPM. The solid and hollow markers are respectively drawn from the experimental data sets and simulated data sets. (**a**) the valence surface is the graph of the equation (1). (**b**) the arousal surface is the graph of the equation (2). (**c**) the gradient field of valence is the gradient field of the valence surface (**a**). (**d**) the gradient field of arousal is the gradient field of the arousal surface (**b**).

To uncover the properties of the affective HMPM, its surfaces are illustrated in the upper panel of Fig. 6(a,b), and their corresponding gradient fields are shown in the lower panel of Fig. 6(c,d). The valence and arousal surfaces obviously describe the nonlinearities of the SCR affective process which is in line with the consensus that affective psychophysiological processes should be nonlinear. The valence factor is sensitive to the gain and decay time constant dimensions because the overall trend of its gradient field (see Fig. 6(c)) are not parallel to any coordinate axis. This means that the valence factor is mainly determined by the two dimensions: gain, which has neural activation intensity meanings, and decay time constant, which has the meanings of skin conductance different pathways^37^,^45^. Since the EDA1 and EDA2 pathways (Model hypothesis in Methods) have their origins in brain’s motivational circuits, this finding may indirectly support that the affective valence is related to the activations of appetitive subcircuits and defensive subcircuits^9^. And yet, the arousal factor is mainly determined by the gain dimension because the overall trend of its gradient field (see Fig. 6(d)) seems to be parallel to the gain axis. This is in line with the prior research^9^ in which the affective arousal may only reflect the activation intensity of brain’s motivational circuits. Hence, the affective HMPM can provide certain indirect evidences that the valence and arousal have their origins in human brain’s motivational circuits.

### Comparison with ANN

**Figure 7:**
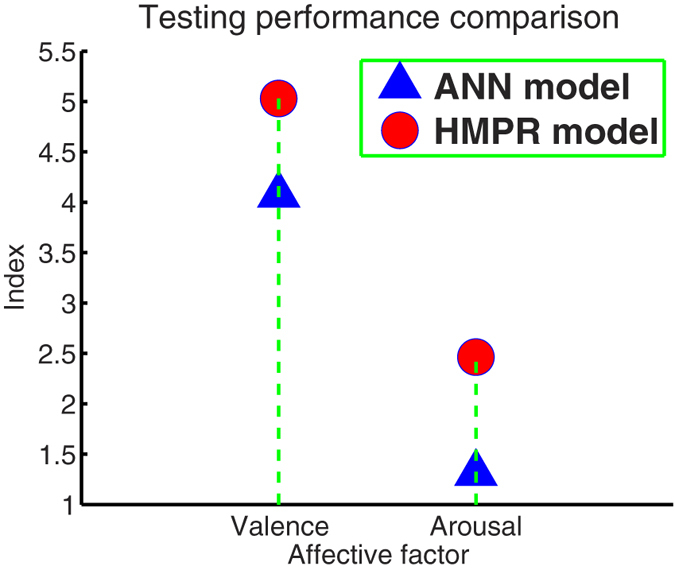
Comparison between the affective HMPM and the optimal ANN models.

In order to further illustrate the effectiveness of the HMPR method, we compared it with the commonly used ANN method. When solving black box modeling problems, people usually use the ANN because every continuous function that maps intervals of real numbers to some output interval of real numbers can be approximated arbitrarily closely by a multilayer perceptron with just one hidden layer^46^,^47^. Using the Neural Network Toolbox in Matlab R2013b and simulated data sets, we trained ANN models that include three layers: two neurons input layer that accept gains and time constants, one hidden layer whose neurons are variable (from 1 to 10 neurons), and one neuron output layer. The performances of these ANN models are listed in the Supplementary Table S6. Its index rows illustrate that the ANN model of nine hidden neurons is the best for estimating valence, and the ANN model of ten hidden neurons is the best for estimating arousal. The final performances of the optimal ANN models on the experimental data sets is that r = 0.9765(0.9534, 0.9865), p-value = 1.4077*e* − 17, MSE = 0.2398, and Index = 4.0726 for valence estimation; and r = 0.9586(0.9473, 0.9634), p-value = 5.1062*e* − 21, MSE = 0.7311, and Index = 1.3111 for arousal estimation (see Supplementary Table S7). Since the correlation coefficients and MSEs of the affective HMPM are higher and lower than those of the optimal ANN model, respectively, the total performance of the affective HMPM is slightly better than that of the optimal ANN model (see Fig. 7). Note that it may be strange that the optimal ANN model performed very well during training process (modeling process), but lost its good performances in the testing process. The reason for this phenomenon is that the ANN is good at learning the local characteristics of training data sets^46^. The performance of the ANN models rapidly increased during the training process, but the final performance of the ANN models may significantly reduced in the testing process. Compared with the ANN, the HMPR is good at learning the whole characteristics of training data sets because its foundations are the addition of polynomial surfaces. The performance of the HMPR is relatively stable in both training and testing processes. Moreover, although correlation coefficients of the affective HMPM are higher than those of the optimal ANN model, there are no significant differences. Hence, the HMPR and ANN both are good methods to obtain accurate estimation, but the HMPR is more intuitive and stable.

## Discussion

Using the mature affective induction experiment and indicative skin conductance signal, we specifically introduced the higher-order multivariable polynomial regression method to efficiently estimate the affective valence and arousal from pure skin conductance responses. The fundamental data sets (see Supplementary Table S1) showed that the pleasant, neutral, and unpleasant affects were successfully induced by our experiment (see Fig. 3). To solve the technical problem of requiring large data sets in carrying out the HMPR, we proposed an ad-hoc statistical formula (equation (4)) to construct the simulated data sets from the experimental data sets. The simulated data sets maintain the patterns of the experimental data sets and contain more data points than those of the experimental data sets (see Fig. 4). The HMPR results yielded three dominating findings. Firstly, we found that a nonlinear model (a HMPM) is often better than a linear model (a MLR model) (see Fig. 5). This was in line with prior researches^5^,^6^,^13^,^14^,^31^,^34^. In the present study, a HMPM can give a better description of nonlinearities of the SCR affective process than what a linear model can do. Secondly, the affective HMPM provides significant correlation coefficients, r = 0.9801 for valence estimation, and r = 0.9600 for arousal estimation (confidence level 0.0001). The affective HMPM visually and accurately stated the nonlinearities of the SCR affective process (see Fig. 6(a,b)). Thirdly, the gradient fields of the affective HMPM (see Fig. 6(c,d)) provided certain indirect evidences that the affective valence and arousal have their origins in human brain’s motivational circuits. Moreover, the result of comparing the HMPR with the ANN illustrate that both HMPR and ANN are good methods to solve the affective estimation problem, and the HMPR is good at learning the whole characteristics of data sets and more intuitive and stable than the ANN.

By using the gradient fields (see Fig. 6(c,d)), the affective HMPM indirectly supports that the affective valence and arousal have their origins in human brain’s motivational circuits. It seems to be reasonable that evaluative affective components (valence and arousal) are associated with the broad functions of brain’s motivational circuits—appetitive subcircuits activation (pleasant) and defensive subcircuits activation (unpleasant) and an intensity of these two subcircuits activation (arousal)^9^. Affective cues can induce skin conductance activations through the limbic-hypothalamic EDA1 pathway, and the pleasant affect may additionally induce skin conductance activations through the premotor-basal ganglia EDA2 pathway^37^. The gradient field of valence (Fig. 6(c)) indicates that the valence factor is mainly determined by the two dimensions: gain, that has neural activation intensity meanings, and decay time constant, that has the meanings of skin conductance different pathways^37^,^45^. The gradient field of arousal (Fig. 6(d)) indicates that arousal factor is mainly determined by the gain dimension. These findings may indirectly support prior researches^9^,^48^.

The HMPR is an important supplement to emotional estimation methodology. The HMPR, in fact, is not only theoretically supported by the Taylor theorem, but also able to obtain an intuitive HMPM to efficiently estimate the affective valence and arousal from pure skin conductance responses. Moreover, the result of comparing the HMPR with the ANN models (see Supplementary Table S7) showed that both the HMPR and ANN can obtain relative accurate computing results. Such accurate estimation results surely increases the impact in the wearable computing fields such as smart watches, Mi Band, and Google Glass, etc. It is a trend now to detect human affect by multimodal signals (e.g., neural activations, facial videos, voice recordings, body gestures, and physiological signals, etc.)^3^,^5^,^12^,^15^,^17^,^18^,^19^,^24^,^26^,^35^,^36^,^49^. The use of the HPMR on the multimodal signals can bring obvious benefits that human affective states can be effectively detected, at the same time, much more detailed psychophysiological and neural mechanisms can be revealed for medical and economic applications, if one can effectively solve the three main open problems that include illustrating certain neural mechanisms, obtaining pure affective signal patterns for each modal, and efficiently fusing these multimodal feature vectors.

## Methods

In this section, the model hypothesis, experiment, and mathematical foundations are listed to support the present study.

### Model hypothesis

Based on the findings in anatomy and neural sciences, we proposed the hypothesis that there exists the implicit complex mapping from pure skin conductance responses (SCRs) to their corresponding affective states. The brain and peripheral organs are connected by efferent and afferent neural fibers and mutually influence each other, although the brain is the control and information processing center^9^,^35^,^36^,^50^. Affective cues activate motivational circuits evolved in the mammalian brain that consists of defense and appetitive subcircuits^9^,^48^. In the motivational circuits, the bilateral amygdalas play a central role: Motivationally relevant cues projected from cortex and thalamus or hippocampus activate the amygdala’s central and lateral nuclei and its extension in the bed nucleus of the stria terminalis^9^. Subsequent projections from these amygdaloid structures engage a variety of other brain regions (e.g., nucleus ambiguous, the dorsal motor nucleus of the vagus, and the hypothalamus) that activate efferent projecting structures that mediate defensive or appetitive reflex actions^9^,^48^,^51^. Although brain defensive and appetitive subcircuits are highly overlapped in their individual neural structures and connectivity, consisting with the broad similarity in autonomous autonomic nervous system (ANS) response^24^, both animal and human research have implicated that the ventral striatum (e.g., nucleus accumbens) and ventral medial prefrontal cortex (VMPFC) are additional neural structures specific to appetitive subcircuits^9^,^52^,^53^. It seems to be reasonable that evaluative affective factors (valence and arousal) are associated with the broad functions of brain’s motivational circuits—appetitive subcircuits activation (pleasant) and defensive subcircuits activation (unpleasant), and an intensity of these two subcircuits activation (arousal)^9^. For the time being, although the complex central origins of skin conductance is still incomplete, the experimental as well as clinical evidence concerning the central nervous system (CNS) elicitation of electrodermal activity (EDA) points to the existence of two different origins, a limbic-hypothalamic source labeled EDA1, being emotionally influenced, and a premotor-basal ganglia source labeled EDA2, eliciting electrodermal concomitants of the preparation of specific motor actions^37^. The ventral striatum, which is specific to appetitive subcircuits and a part of basal ganglia, and VMPFC can elicit skin conductance activity by the EDA2 pathway. Moreover, the responses of peripheral systems are very important for people to experience affect. In a word, interactions between brain and peripheral organs and the overlapped CNS mechanisms of affect and skin conductance ensure the rationality of the hypothesis.

### Experiment

The experimental protocol for this study was approved by the local ethics committee of the Southwest University and an informed consent was obtained from every subject involved in the experiment. The methods were carried out in accordance with the approved guidelines.

#### Two dimensional affective model

Since the estimation problem in the AD was studied, we adopted the most widely used valence-arousal model (the Circumplex Model of Affects (CMA)) to continuously represent subjects’ affective states^6^,^18^,^54^,^55^,^56^. The CMA model considers two continuous dimensions, in which the valence (V) dimension represents how much an affect is perceived as pleasant or unpleasant (ranging from unpleasant to pleasant), whereas the arousal (A) dimension indicates how strongly the affect is felt (ranging from calm to excited). According to the work of Lang *et al.*^44^,^47^,^57^,^58^, we used the Self-Assessment Manikin (SAM) to assess these two affective scores on a scale from 1 to 9.

#### Subjects

Thirty second-year undergraduate healthy female subjects (mean age = 19.44; SD = 1.09; range = 18–22) not suffering from evident mental pathologies, were recruited to participate in the affective experiment. Since previous behavioral and brain researches revealed that women show a great sensitivity to negative information compared to men^44^,^59^,^60^,^61^, the study chose the female subjects to guarantee the validity of the experiment. To control the influence of menstrual cycle on processing negative emotions^62^,^63^, they took participate in the experiment out of their menstrual cycles. The subjects reported no history of affective disorder and were free of any psychiatric medication. The subjects were affectively healthy, indicated by the low scores in the Spielberger trait and state anxiety scales (total = 80 for either scale). The averaged trait, state anxiety scores were 33.27 (SD = 1.24) and 33.73 (SD = 1.12), respectively. All subjects are right-handed, with normal or corrected-to-normal visual acuity. Because of operation errors of experimenters in the first day, 27 subjects’ data were included in the study.

#### Stimuli

As the International Affective Picture System (Peter J. Lang *et al.*^51^,^58^,^59^) is one of most frequently cited tools in the area of affective elicitation^6^,^20^, and allows better event-related experimental control, the study selected pictures from it as experimental stimuli. The IAPS provides a set of normative colored pictures to induce affective states. The standard affective ratings of IAPS pictures were obtained with the easier adapted 9-point SAM scale^58^. Based on the valence and arousal ratings obtained from a prior validation experiment, and the cultural differences between Chinese and English speaking people, twenty four pictures, depicting 8 pleasant, 8 neutral, and 8 unpleasant ones, were chosen. The slide numbers of selected pictures are the following: (a) pleasant: 1710, 1722, 7230, 7260, 7270, 7330, 7460, 8500; (b) neutral: 5510, 5530, 5740, 7000, 7004, 7006, 7010, 7020; and (c) unpleasant: 3010, 3030, 3053, 3060, 3071, 3080, 3102, 3120. In order to train subjects to be familiar with the experimental process, three additional neutral pictures (slide No., 5300, 5870 and 7580) were also selected.

#### Design

**Figure 8:**
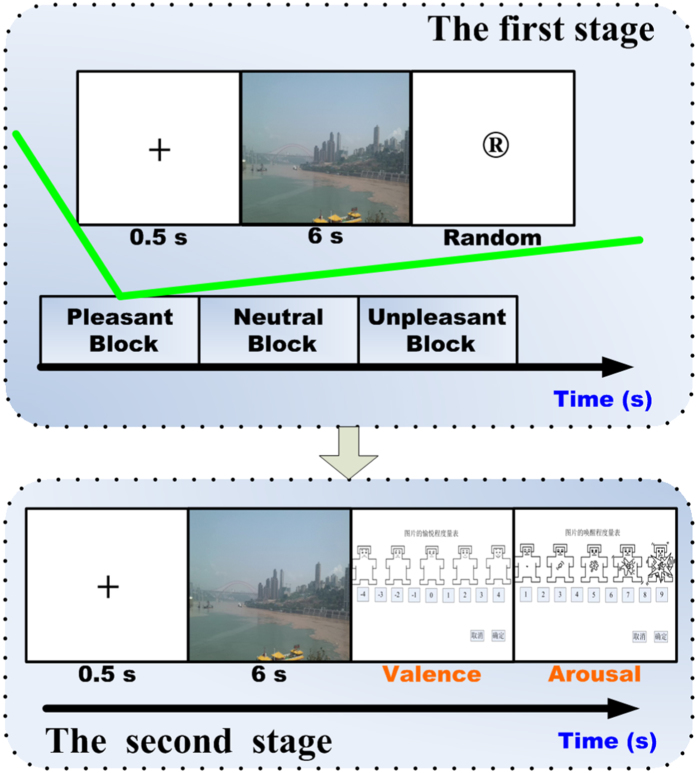
Experimental Design. In the first stage, experimental stimuli are grouped in to pleasant, neutral, and unpleasant blocks. Each block contains eight pictures. For each trial, after a 0.5 s fixation the picture presentation last for 6 s and a random rest followed (29 s, 31 s and 34 s). In the second stage, subjects were required to rate pictures’ valence and arousal by the SAM scales.

The study adopted the event-related experimental design. subjects were required to passively observe affective pictures in the first stage and then rate these pictures in the second stage after the 10 minutes rest (see Fig. 8). In the first stage, we presented pleasant, neutral, and unpleasant blocks in sequence, and four-channel signals were synchronously recorded by the Biopac MP 150 system. In each block consisting of 8 pictures, picture was presented in a random order in the center of the monitor in the testing room for 6 s, with a 0.5 s fixation and a random rest time (29 s, 31 s and 34 s). subjects were required to watch each picture during the entire time of exposition and try to avoid unnecessary body movements. The recorded signals were environmental temperature, Pulse, SC, ECG, and subject’s facial videos. After 10 minutes rest, subjects were required to rate pictures. With a 0.5 s fixation and 6 s picture presentation, subjects immediately rated pictures that were presented at the complete random order by clicking the corresponding buttons in the SAM scales^44^,^47^,^57^,^58^ on screen. The valence and arousal scores of each picture were automatically recorded and converted. The particular details of establishing affective data acquisition system and signal acquisition settings are stated in the Supplementary Affective data acquisition system and settings where the Psychophysics Toolbox Version 3^64^,^65^ and “standard methodology” for SC^66^ are used.

### Mathematical foundations

Here, four mathematical foundations are stated. They are the Lim’s nonlinear curve fitting to extract pure affective SCRs, a statistical formula to construct the simulated data sets, the HMPR to approximate the assumed implicit complex mapping relationship that translates pure SCRs into their corresponding affective states, and model evaluation metrics to choose and evaluate models.

#### Lim’s nonlinear curve fitting

In 1997, the nonlinear curve fitting was used by Lim *et al.* to decompose event-related skin conductance signals into their tonic and phasic components (SCLs and SCRs)^45^. The pure SCR waveform can be mathematically described by a four-parameter function model as follows:


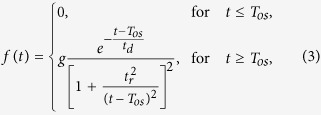


where the four parameters are: *g* is gain, being related with the neural activation intensity; *T*_*os*_ is response onset time, indicating its time latency after a stimulus; *t*_*r*_ is rise time, indicating its rising slope pattern, and *t*_*d*_ is decay time constant, indicating its decay pattern. Following this pure SCR model (equation (3)), Lim *et al.* used a six-parameter SC model to describe typical SC signals that has a SCR occurring on a decaying limb of a previous response by adding a term 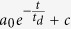 to *f*(*t*), where *a*_0_ is the initial value of the SC signal at stimulus onset and *c* represents the tonic constant (SCL). Given an event-related typical SC signal, the tail of the previous response 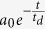, one SCR *f*(*t*), and the SCL *c* are successfully separated by the standard iterative non-linear least-squares routine known as the Marquardt-Levenberg method^45^.

#### A statistical formula

A statistical formula is stated in the following theorem whose analytical derivations are seen in the Supplementary Proof of the data extending theorem. Applying the statistical formula, one can construct a new random variable set from a sample of a population. The size of the new set is 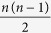 if the size of the sample is *n*.

**Theorem 1 (Data extending theorem)** *Let ξ*_1_, *ξ*_2_, …, *ξ*_*n*_ *be a sample of a population ξ with a finite mean μ and variance σ*^2^*, then*

1) *The new random variables*


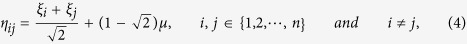


*have the mean μ and variance σ*^2^;

2) *The correlation coefficient of any two different random variables, η*_*ij*_ *and η*_*kl*_, *i*, *j*, *k*, 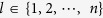, *is* 0 *or* 0.5;

3) *The correlation coefficient of any two different random variables, η*_*ij*_ *and ξ*_*k*_, *i*, *j*, 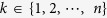, *is* 0 *or* 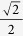.

#### Higher-order Multivariable Polynomial Regression

To accurately approximate a multivariable smoothing function on experimental observations, the HMPR method is a good choice because of the Taylor theorem. For a single-output static system that is governed by a *m*-variable smooth function 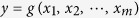, the *p*^*th*^-order multivariable polynomial function 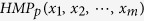,


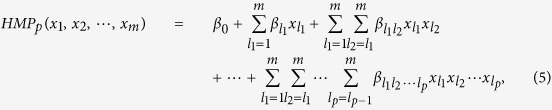


can be used to approximate the smooth function 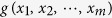 at any precision no matter how complex the function 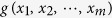 is, where 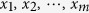 is the input variables of the system, the order *p* is a certain nonnegative integer, and 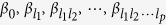 are polynomial coefficients. Noting that the multivariable function 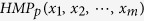 (equation (5)) is a linear one with respect to its polynomial coefficients, we can adopt the standard linear least square estimation (LSE) to find these polynomial coefficients from experimental observations.

Using the HMPR to model a static system, it should be noted that there are two main technical problems: requiring big data sets and matrix being singular. In order to obtain an accurate HMPM, it is often to choose a relative high order polynomial that has many polynomial coefficients to be estimated. This means that many training data points are necessary. To solve the problem of requiring big data sets, we adopted a technology solution: using equation (4) to construct the simulated data sets, training HMPMs on the simulated data sets, and verifying the obtained HMPM on the entire experimental data sets. This technology solution would obtain the relative stable performance of the obtained HMPM because testing sets are the entire experimental data sets. Since the nonlinear terms 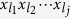 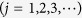 may result in the occurrence of a numerical singular matrix, the singular value decomposition (SVD) and the truncated Least-Squares estimation (tLS) methods are used^67^. The particular details are seen in the Supplementary Particular details on implementing HMPR.

#### Model evaluation metrics

In order to evaluate the performance of an estimation model, we adopt the Pearson’s correlation coefficient (r) and the mean squared error (MSE) that are the most commonly used evaluation metrics^5^,^29^, and proposed an index to choose and evaluate computational models. Given an model, the r between experimental observations and model predicted values is defined as follows:


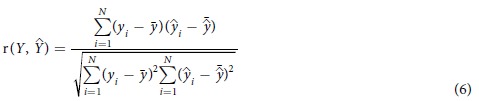


where the variable 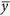, 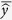 are means of the experimental observation 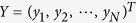 and model prediction 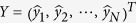, respectively. The MSE of the model is defined as follows:


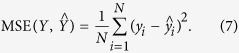


In general, the more accurate the model is, the bigger the r is and the smaller the MSE is. By combining r and MSE metrics, the Index is defined as follows:


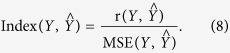


Hence, the better the model is, the bigger the Index is.

## Additional Information

**How to cite this article**: Wei, J. *et al.* Higher-order Multivariable Polynomial Regression to Estimate Human Affective States. *Sci. Rep.* **6**, 23384; doi: 10.1038/srep23384 (2016).

## Supplementary Material

Supplementary Information

## Figures and Tables

**Figure 1:**
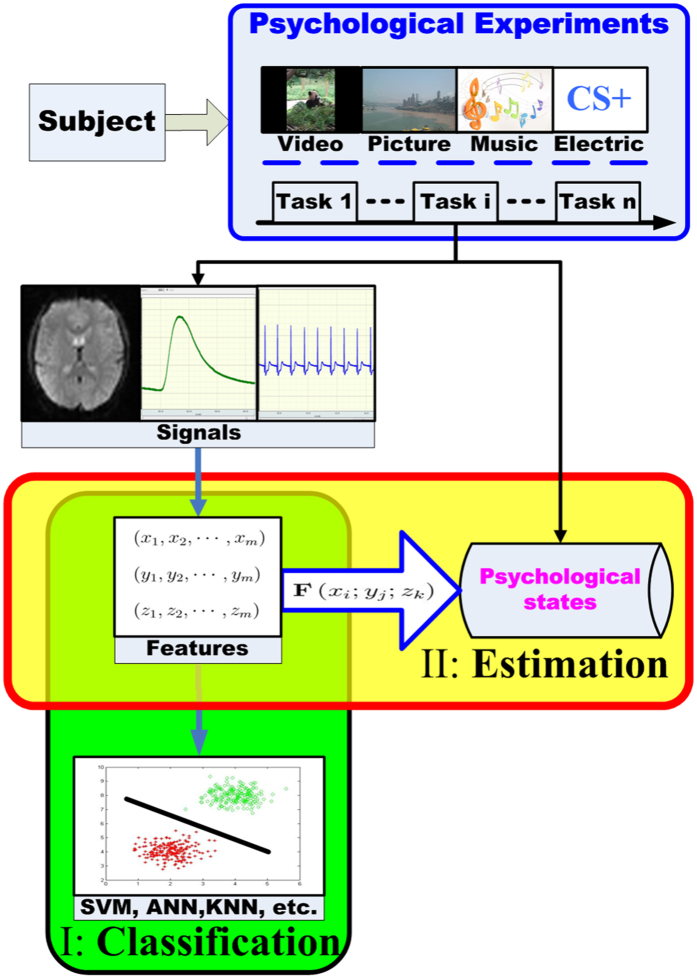
A graphical representation of two main directions in current AD researches. A subject takes participate in various affective psychophysiological experiments in which video, picture, music and electric stimuli are commonly used to induce subject’s different affective states. For each task, various kinds of signals are recorded by physical equipments (e.g., functional magnetic resonance imaging (fMRI) machines, physiological signal acquisition systems, cameras, etc.) on the one hand; and on the other hand, the corresponding affective states are measured according to psychological scales and inventories. Starting from these observed signals, feature vectors are extracted, used to represent response patterns of different affective states, and classified into finite classes by using various classifiers (e.g., support vector machine (SVM), artificial neural network (ANN), k-nearest neighborhood (KNN), etc.) to realize the classification of affective states. Applying the mapping relationships between observed response patterns and the corresponding affective psychological states, researchers use function approximation methods (e.g., multivariate linear-regression analysis, partial least-square estimation, support vector regression, artificial neural network, fuzzy logical analysis, and sequence Bayessian analysis) to approximate the assumed function models (**F**(*x*_*i*_; *y*_*j*_; *z*_*k*_)). Affective states are estimated by these obtained function models and new observed response patterns.

**Figure 2:**
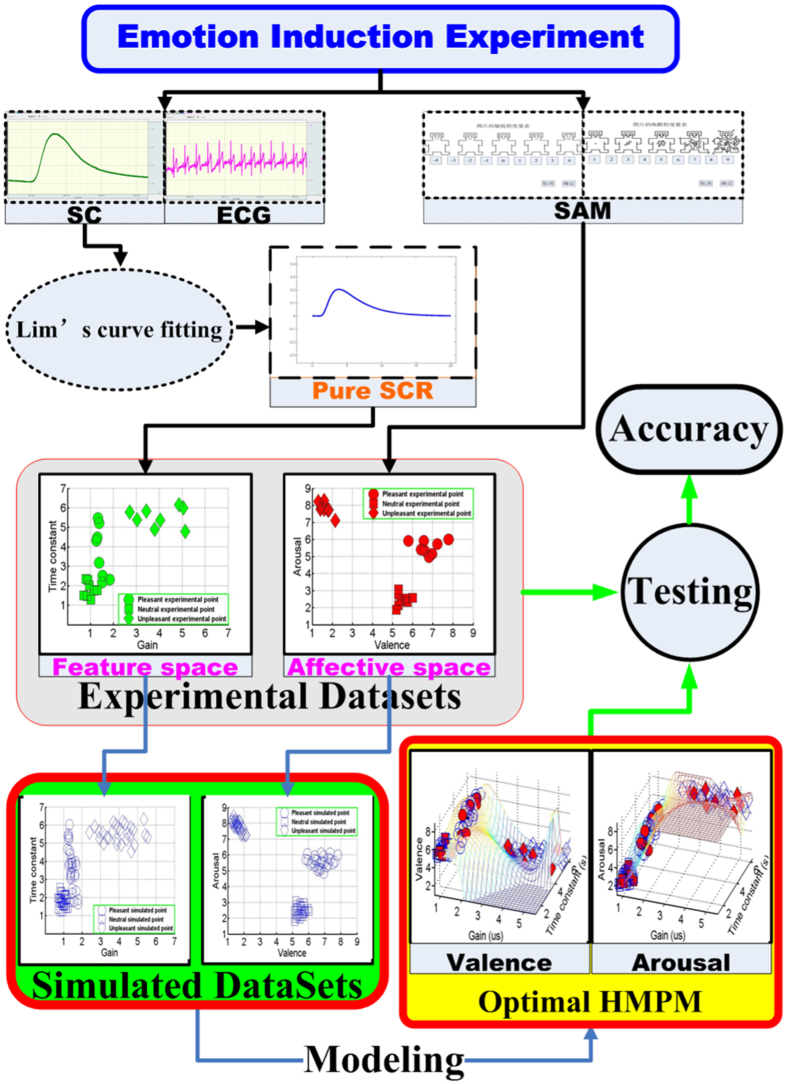
A general overview of the proposed estimation method and block scheme of the overall signal processing. The subjects were emotionally stimulated through pictures chose from the International Affective Picture System (IAPS). For each trial including the 0.5 seconds fixation, 6 seconds picture presentation, and random rest (29, 31, and 34 seconds), physiological signals (skin conductance (SC), electrocardiogram (ECG), and pulse) were recorded by the Biopac MP150 system and the scores of valence and arousal were measured according to the Self-Assessment Manikin (SAM) developed by Margaret M. Bradley and Peter J. Lang^57^. Starting from average skin conductance segments, pure skin conductance response (SCR) patterns were acquired by using the Lim’s curve fitting^45^,^68^. The features (gain and decay time constant (Time constant)) were extracted from these pure skin conductance responses and formed the two dimensions of the physiological feature space. Valence and arousal factors form the two dimensions of the affective space. These experimental gains, decay time constants, and affective scores form the experimental data sets. Based on the experimental data sets, the simulated data sets were statistically constructed by equation (4) in the methods section. Then, the optimal affective HMPM were established on the simulated data set. Finally, the obtained HMPM were tested on the entire experimental data sets to evaluate its accuracy.
