# Supplementary material for: Higher-order Multivariable Polynomial Regression to Estimate Human Affective States
Source: Sci Rep. 2016 Mar 21;6:23384. doi: 10.1038/srep23384 (PMC4800309; doi:10.1038/srep23384)
Supplement: Supplementary Information [file srep23384-s1.pdf]

Supplementary Information for Scientific Reports

Higher-order Multivariable Polynomial Regression to Estimate Human Affective States

Jie Wei[1,2,+], Tong Chen[1,2,+], Guangyuan Liu[1,2,*], and Jiemin Yang[3]

[1]School of Electronic and Information Engineering, Southwest University, Chongqing, 400715, PR China
[2]Chongqing Key Laboratory of Non-linear Circuit and Intelligent Information Processing, Southwest University, Chongqing, 400715, PR China
[3]Faculty of Psychology, Southwest University, Chongqing, 400715, PR China
[*]corresponding.liugy@swu.edu.cn
[+]These authors contributed equally to this work

Affective type	Slide No.	Physiological SCR pattern				Affective pattern	
		Onset time	Gain	Rise time	Time constant	Valence	Arousal
Pleasant	1710	0.86(0.07)	1.38(0.17)	1.62(0.16)	3.18(0.40)	7.78(1.35)	6.00(2.16)
	1722	1.05(0.03)	1.52(0.10)	1.30(0.05)	2.18(0.06)	5.78(2.01)	5.93(1.75)
	7230	0.52(0.15)	1.29(0.09)	1.08(0.19)	4.48(0.51)	7.22(1.45)	5.74(2.12)
	7260	0.95(0.16)	1.32(0.93)	2.37(0.45)	5.48(0.43)	6.81(2.21)	4.96(1.95)
	7270	0.91(0.35)	1.26(0.67)	1.85(0.44)	4.32(0.20)	6.59(1.54)	5.33(1.96)
	7330	1.18(0.17)	1.38(0.51)	1.74(0.34)	5.22(0.93)	6.56(1.98)	5.93(1.96)
	7460	1.00(0.80)	1.83(0.22)	0.82(0.21)	2.32(0.81)	6.96(2.12)	5.15(2.05)
	8500	1.02 (0.04)	1.53(0.23)	1.23(0.09)	2.51(0.12)	6.41(1.36)	5.41(1.88)
Neutral	5510	1.03(0.22)	1.15(0.09)	1.10(0.05)	1.75(0.05)	5.30(1.42)	2.77(2.11)
	5530	0.85(0.14)	0.89(0.10)	1.23(0.07)	2.30(0.12)	5.30(1.43)	3.06(1.95)
	5740	1.22(0.23)	0.80(0.12)	1.26(0.09)	2.36(0.24)	6.00(1.41)	2.58(2.31)
	7000	1.10(0.23)	1.30(0.25)	1.36(0.12)	1.77(0.14)	5.30(1.33)	2.14(1.83)
	7004	1.13(0.13)	0.95(0.11)	1.50(0.09)	2.01(0.10)	5.74(1.19)	2.32(1.72)
	7006	1.14(0.15)	0.85(0.23)	1.21(0.20)	1.48(0.15)	5.63(1.18)	2.54(2.17)
	7010	1.10(0.25)	1.02(0.31)	1.20(0.18)	1.29(0.78)	5.41(1.21)	2.40(2.09)
	7020	1.14(0.03)	0.71(0.12)	1.24(0.11)	1.51(0.12)	5.19(1.17)	1.88(1.78)
Unpleasant	3010	0.14(0.09)	4.85(0.13)	2.10(0.42)	6.18(0.30)	1.44(0.69)	7.93(1.14)
	3030	0.30(0.12)	5.05(0.74)	2.03(0.46)	6.01(0.89)	2.15(0.94)	7.11(1.39)
	3053	0.77(0.15)	3.43(0.19)	1.45(0.16)	5.84(0.26)	1.59(1.22)	8.30(1.23)
	3060	0.82(0.13)	3.03(0.16)	1.27(0.05)	5.39(0.29)	1.30(0.64)	8.22(1.12)
	3071	0.93(0.24)	5.13(0.21)	1.62(0.12)	4.79(0.26)	1.60(0.79)	7.78(1.28)
	3080	0.26(0.12)	3.79(0.05)	1.66(0.24)	4.89(0.15)	1.41(0.73)	7.78(1.12)
	3102	1.00(0.21)	2.71(0.06)	0.98(0.03)	5.78(0.16)	1.78(0.80)	7.74(1.09)
	3120	0.56(0.16)	4.04(0.51)	2.09(0.13)	5.37(0.51)	1.81(0.92)	7.74(1.25)

Supplementary Table 1. Affective pattern and psychological SCR pattern. Twenty four pictures, which are defined by their slide numbers (Slide No.) in the IAPS, were grouped into pleasant, neutral, and unpleasant groups. For each picture, its affective pattern contains its mean valence and arousal scores across participants. Its mean SC signal is obtained by averaging 10-second SC segments after its presentation across participants. Applying the Lim's nonlinear curve fitting with 20 step iterations to each mean SC signal, each pure SCR pattern (waveform) was represented by a four dimensional feature vector that consists of onset time, gain, rise time, and decay time constant (Time constant) parameters. The values in the parentheses are corresponding standard deviations.

Affective type	Physiological SCR dimension		Affective pattern	
	Gain	Time constant	Valence	Arousal
Pleasant	1.45	2.25	6.78	6.13
	1.29	3.88	7.80	6.00
	1.31	4.58	7.52	5.45
	1.27	3.77	7.36	5.71
	1.35	4.40	7.33	6.13
	1.67	2.35	7.62	5.58
	1.46	2.49	7.23	5.77
	1.39	3.17	6.39	5.95
	1.41	3.88	6.10	5.40
	1.37	3.06	5.95	5.66
	1.45	3.69	5.92	6.08
	1.77	1.64	6.21	5.53
	1.56	1.78	5.81	5.71
	1.25	5.50	7.12	5.27
	1.21	4.69	6.97	5.53
	1.29	5.32	6.94	5.95
	1.61	3.27	7.23	5.40
	1.40	3.41	6.84	5.58
	1.23	5.39	6.68	4.98
	1.31	6.03	6.65	5.40
	1.63	3.98	6.94	4.85
	1.42	4.11	6.55	5.03
	1.27	5.21	6.50	5.66
	1.59	3.16	6.78	5.11
	1.37	3.29	6.39	5.29
	1.67	3.80	6.76	5.53
	1.46	3.93	6.36	5.71
	1.78	1.88	6.65	5.16
Neutral	1.05	2.11	5.22	3.10
	0.98	2.16	5.72	2.76
	1.34	1.74	5.22	2.45
	1.09	1.91	5.53	2.58
	1.02	1.53	5.46	2.74
	1.13	1.40	5.30	2.63
	0.92	1.55	5.14	2.26
	0.80	2.54	5.72	2.97
	1.16	2.13	5.22	2.66
	0.91	2.30	5.53	2.79
	0.84	1.92	5.46	2.95
	0.95	1.79	5.30	2.84
	0.74	1.94	5.14	2.47
	1.09	2.17	5.72	2.32
	0.84	2.34	6.03	2.45
	0.78	1.96	5.95	2.61
	0.89	1.83	5.80	2.50
	0.67	1.98	5.64	2.13
	1.20	1.92	5.53	2.13
	1.13	1.55	5.46	2.29
	1.24	1.42	5.30	2.19
	1.03	1.57	5.14	1.82
	0.88	1.72	5.77	2.42
	0.99	1.59	5.61	2.32

Continued on next page

Continued from previous page

	0.78	1.74	5.46	1.95
	0.92	1.21	5.53	2.47
	0.71	1.36	5.38	2.11
	0.82	1.23	5.22	2.00
Unpleasant	5.34	6.32	1.86	7.39
	4.20	6.20	1.47	8.23
	3.92	5.88	1.26	8.18
	5.40	5.46	1.47	7.86
	4.46	5.53	1.34	7.86
	3.69	6.16	1.60	7.84
	4.63	5.87	1.63	7.84
	4.34	6.09	1.97	7.65
	4.06	5.77	1.76	7.60
	5.54	5.34	1.97	7.29
	4.60	5.42	1.84	7.29
	3.83	6.04	2.10	7.26
	4.77	5.75	2.13	7.26
	2.91	5.65	1.37	8.44
	4.39	5.23	1.58	8.13
	3.45	5.30	1.44	8.13
	2.68	5.93	1.71	8.10
	3.62	5.64	1.73	8.10
	4.11	4.91	1.37	8.07
	3.17	4.98	1.23	8.07
	2.40	5.60	1.50	8.05
	3.34	5.32	1.52	8.05
	4.65	4.56	1.44	7.76
	3.88	5.18	1.71	7.73
	4.83	4.89	1.73	7.73
	2.94	5.25	1.58	7.73
	3.88	4.97	1.60	7.73
	3.11	5.59	1.86	7.71

Supplementary Table 2. Simulated data sets. For each affect and column, twenty eight simulated values were constructed from its experimental values by the data extending theorem (Theorem 1).

Polynomial order	Valence						Arousal					
	PCOR				MSE	Index	PCOR				MSE	Index
	r	p	LB	UB			r	p	LB	UB		
1	0.85	0.00	0.78	0.90	1.31	0.65	0.91	0.00	0.87	0.94	0.83	1.10
2	0.91	0.00	0.86	0.94	0.83	1.09	0.96	0.00	0.94	0.97	0.38	2.55
3	0.97	0.00	0.95	0.98	0.31	3.08	0.96	0.00	0.94	0.97	0.40	2.40
4	0.97	0.00	0.95	0.98	0.30	3.21	0.97	0.00	0.95	0.98	0.34	2.88
5	0.97	0.00	0.96	0.98	0.25	3.91	0.97	0.00	0.95	0.98	0.33	2.94
6	0.96	0.00	0.96	0.97	0.30	3.16	0.96	0.00	0.95	0.98	0.35	2.79

Supplementary Table 3. Performances of twelve models with three input variables on the simulated data sets. The PCOR and MSE are respectively the Pearson's correlation and mean squared error (equations (6) and (7)) of models prediction. Under the PCOR column, r is the Pearson's correlation coefficient, p is the p-value, and LB and UB are respectively the lower and upper bounds of the 95% confidence interval of r. The Index is the ratio of model's MSE to correlation coefficient r (equation (8)). All p-values are no more than 0.0001.

The five order HMPMs with three inputs is as follows:

$$\begin{cases} \mathrm{V}(T,g,t_d) = & 2.4472+3.5244g^2+2.6252gt_d-1.2297g^3-2.9242g^2t_d+0.9119g^3t_d+0.0201g^5 \\ & -0.0857g^4t_d+\varepsilon_V, \\ \mathrm{A}(T,g,t_d) = & -0.8618+2.9013gt_d+0.35t_d^2-0.9844gt_d^2+0.0992gt_d^3-0.0076t_d^4-0.0021g^2t_d^3+\varepsilon_A, \end{cases} \quad (1)$$

where T, g, and t_d are onset time, gain, and time constant, and the terms $\varepsilon_V, \varepsilon_A$ are errors.

Polynomial order	Valence						Arousal					
	PCOR				MSE	Index	PCOR				MSE	Index
	r	p	LB	UB			r	p	LB	UB		
1	0.85	0.00	0.78	0.90	1.31	0.65	0.91	0.00	0.87	0.94	0.83	1.10
2	0.91	0.00	0.86	0.94	0.83	1.09	0.96	0.00	0.95	0.98	0.34	2.83
3	0.97	0.00	0.95	0.98	0.33	2.89	0.96	0.00	0.94	0.97	0.40	2.40
4	0.97	0.00	0.95	0.98	0.31	3.11	0.97	0.00	0.95	0.98	0.30	3.20
5	0.98	0.00	0.97	0.99	0.16	6.15	0.97	0.00	0.95	0.98	0.31	3.11
6	0.98	0.00	0.96	0.98	0.22	4.37	0.96	0.00	0.95	0.98	0.34	2.83
7	0.97	0.00	0.96	0.98	0.25	3.97	0.97	0.00	0.95	0.98	0.32	3.01
8	0.98	0.00	0.98	0.99	0.15	6.73	0.96	0.00	0.94	0.98	0.35	2.73
9	0.98	0.00	0.96	0.98	0.24	4.11	0.97	0.00	0.96	0.98	0.28	3.42
10	0.98	0.00	0.96	0.99	0.24	4.00	0.96	0.00	0.94	0.97	0.38	2.52

Supplementary Table 4. Performances of twenty models with two input variables on the simulated data sets. The PCOR and MSE are respectively the Pearson's correlation and mean squared error (equations (6) and (7)) of models prediction. Under the PCOR column, r is the Pearson's correlation coefficient, p is the p-value, and LB and UB are respectively the lower and upper bounds of the 95% confidence interval of r. The Index is the ratio of model's MSE to correlation coefficient r (equation (8)). All p-values are no more than 0.0001.

Valence model				Arousal model			
Parameter	value	LB	UB	Parameter	value	LB	UB
α_{00}	4.64304351	4.55186803	4.73421900	β_{00}	1.09673702	0.97239774	1.22107630
α_{40}	-1.13887275	-1.62341059	-0.65433491	β_{21}	1.63403427	1.31146025	1.95660829
α_{31}	2.40514460	1.63864756	3.17164164	β_{22}	-0.53044020	-0.63966746	-0.42121293
α_{32}	-1.16460227	-1.49501333	-0.83419121	β_{05}	0.02478456	0.01894402	0.03062511
α_{14}	0.05101483	0.03935123	0.06267842	β_{24}	0.01300670	0.01030580	0.01570760
α_{42}	0.35987576	0.23750260	0.48224892	β_{06}	-0.00801417	-0.01020255	-0.00582578
α_{06}	-0.00061845	-0.00079847	-0.00043844	β_{43}	-0.00188695	-0.00246411	-0.00130978
α_{52}	0.00415980	0.00281086	0.00550874	β_{07}	0.00064024	0.00043599	0.00084449
α_{43}	-0.12418913	-0.16238427	-0.08599399	β_{44}	0.00087617	0.00061029	0.00114205
α_{34}	0.09057930	0.06664330	0.11451530	β_{35}	-0.00050624	-0.00063949	-0.00037299
α_{25}	-0.02145223	-0.02720084	-0.01570362	β_{54}	-0.00003533	-0.00004826	-0.00002241
α_{44}	0.00959053	0.00654917	0.01263189				
α_{35}	-0.00940102	-0.01207745	-0.00672460				
α_{26}	0.00249513	0.00176784	0.00322242				

Supplementary Table 5. Parameters of the final optimal HMPM. LB and UB are respectively the lower and upper bounds of the 95% confidence interval of corresponding parameter.

Hidden neuron	Valence						Arousal					
	PCOR				MSE	Index	PCOR				MSE	Index
	r	p	LB	UB			r	p	LB	UB		
1	0.96	0.00	0.94	0.97	0.40	2.39	0.97	0.00	0.95	0.98	0.31	3.17
2	0.99	0.00	0.98	0.99	0.11	9.02	0.99	0.00	0.98	0.99	0.13	7.48
3	0.99	0.00	0.98	0.99	0.11	9.32	0.99	0.00	0.98	1.00	0.09	10.83
4	0.99	0.00	0.98	0.99	0.10	10.06	0.99	0.00	0.98	1.00	0.08	12.47
5	0.99	0.00	0.99	1.00	0.08	11.98	0.99	0.00	0.99	1.00	0.07	13.80
6	0.99	0.00	0.99	1.00	0.05	20.11	0.99	0.00	0.99	1.00	0.06	16.91
7	0.99	0.00	0.99	0.99	0.08	12.62	0.99	0.00	0.99	1.00	0.06	17.90
8	0.99	0.00	0.99	1.00	0.05	18.33	0.99	0.00	0.99	0.99	0.07	13.68
9	1.00	0.00	0.99	1.00	0.04	25.19	0.99	0.00	0.99	1.00	0.06	16.69
10	1.00	0.00	0.99	1.00	0.04	24.32	1.00	0.00	0.99	1.00	0.05	21.86

Supplementary Table 6. Performances of twenty ANN models with two input variables on the simulated data sets. The PCOR and MSE are respectively the Pearson's correlation and mean squared error (equations (6) and (7)) of models prediction. Under the PCOR column, r is the Pearson's correlation coefficient, p is the p-value, and LB and UB are respectively the lower and upper bounds of the 95% confidence interval of r. The Index is the ratio of model's MSE to correlation coefficient r (equation (8)). All p-values are no more than 0.0001.

Model	Valence						Arousal					
	PCOR				MSE	Index	PCOR				MSE	Index
	r	p	LB	UB			r	p	LB	UB		
HMPR	0.9801	0.00	0.9538	0.9915	0.1948	5.0301	0.9600	0.00	0.9084	0.9828	0.3894	2.4654
ANN	0.9765	0.00	0.9534	0.9865	0.2398	4.0726	0.9586	0.00	0.9473	0.9634	0.7311	1.3111

Supplementary Table 7. Comparison between the affective HMPM and the optimal ANN model on the experimental data sets. The PCOR and MSE are respectively the Pearson's correlation and mean squared error (equations (6) and (7)) of models prediction. Under the PCOR column, r is the Pearson's correlation coefficient, p is the p-value, and LB and UB are respectively the lower and upper bounds of the 95% confidence interval of r. The Index is the ratio of model's MSE to correlation coefficient r (equation (8)). All p-values are no more than 0.0001.

Affective data acquisition system and settings

Affective data acquisition system

The stimulus presentation, acquisition of psychological signals, and subject facial video recordings were synchronously completed by running Psychophysics Toolbox Version 3 (developed by David Brainard, Denis Pelli, and Mario Kleiner, See http://psychtoolbox.org/) and customer scripts on a personal computer. The computer has a four-head graphics card, one head for two monitors (23.6-in.) that were used to present the picture stimuli and were respectively placed in the testing and control rooms, one head for displaying acquired psychological signals, one head for displaying subject facial video recordings, and the last head for displaying the control processes in the command window of the MATLAB 2013a (The Mathworks, Inc, Protected by U.S. and international patents, See http://www.mathworks.com/patents). Experimenters can control the entire experimental process, and simultaneously monitor the experimental stimulus presentation, acquired psychological signals, and subject facial video recordings in the control room. By clicking the corresponding buttons in the SAM scales on the testing room's screen, the valence and arousal scores were automatically recorded and converted into the standard scores from 1 to 9.

Signal acquisition settings

In the first stage, participants were seated in a dimly lit separate testing room, facing a computer monitor that was used to present picture stimuli and placed 100 cm from the participant. Pictures were grouped into pleasant, neutral, and unpleasant blocks. We orderly presented pleasant, neutral, and unpleasant blocks and there are no additional time gaps between the adjacent blocks. In each block consisting of 8 pictures, picture was presented in a random order in the center of the monitor in the testing room for 6s, with a 0.5s fixation and a random rest time (29s, 31s, and 34s). Participants were required to watch each stimulus during the entire time of exposition and try to avoid unnecessary body movements. During the watching process, the environmental temperature, pulse, SC, and ECG signals were recorded by Biopac MP 150 system (Biopac Systems Inc., USA, See http://www.biopac.com/), and subject facial videos were recorded by a common USB webcam. For recording SC (electrodermal activity or galvanic skin response) signal, the "standard methodology" was used. Before attaching the electrodes, the skin was cleaned with alcohol. The electrodes (EL507) for SC were attached to the distal phalanges of index and middle fingers of the left hand. The signal was filtered by the low-pass (LP) filter at 10 Hz for motor, ocular and biological artefacts and the high-pass (HP) filter at direct current (DC). ECG was recorded continuously in terms of R-wave modal from three electrodes (lead I), two of which were attached to the lower wrist, with the positive pole on the left arm and the negative pole on the right one. The ground electrode was placed over the right ankle. The ECG signal was filtered by LP 35Hz and HP 0.05Hz filters with the Biopac Acknowledge 4.2 software according to the manufacturer MP Hardware Guide. The environmental temperature were recorded by placing the BN-TEMP-A-XDCR temperature transducer in the testing room whose temperature and relative humidity were kept at about 24 degree centigrade and 50% by an air condition, respectively. The recorded environmental temperature were filtered by LP 10 Hz and HP 0.5Hz filters. All physiological signal channels were sampled at 1000 Hz and additionally filtered by the 50 Hz notch filter to wipe off the influence of the wall-power line frequency. In order to make participants to be familiar with the experimental process, three training pictures were presented before the start of presenting the pleasant block. During the training segment, no signals were recorded.

After the first stage, participants were required to rest for 10 minutes and then entered into the second stage to rate pictures that were viewed. Before the start of formal rating process, three training pictures were presented and no scores were recorded. With a 0.5s fixation and 6s picture presentation, participants immediately rated each picture that was presented at the complete random order by clicking the corresponding buttons in the SAM scales. The valence and arousal ratings were automatically recorded and converted into the standard range from 1 to 9.

Proof of the data extending theorem

Proof 1 *Since $\xi_1, \xi_2, \cdots, \xi_n$ be a sample of a population ξ with the finite mean μ and variance σ^2, $\xi_1, \xi_2, \cdots, \xi_n$ is an independent random variable sequence, and*

$$E\{\xi_j\} = \mu, \quad D\{\xi_j\} = \sigma^2, \quad (j \in \{1,2,\cdots,n\})$$

where E and D denote the mathematical expectation and variance operators respectively.

For any $i, j \in \{1,2,\cdots,n\}$ and $i \neq j$, we have computations as follows:

$$E\{\eta_{ij}\} = E\{\frac{\xi_i + \xi_j}{\sqrt{2}} + (1-\sqrt{2})\mu\} = \frac{E\{\xi_i\} + E\{\xi_j\}}{\sqrt{2}} + (1-\sqrt{2})\mu = \sqrt{2}\mu + (1-\sqrt{2})\mu = \mu$$

and

$$D\{\eta_{ij}\} = D\{\frac{\xi_i + \xi_j}{\sqrt{2}} + (1-\sqrt{2})\mu\} = \frac{D\{\xi_i\} + D\{\xi_j\}}{2} + 0 = \sigma^2.$$

For any two different random variables, η_{ij} and $\eta_{kl}, i, j, k, l \in \{1,2,\cdots,n\}$, random variables ξ_i, ξ_j are used to compute the random variable η_{ij} and random variables ξ_k, ξ_l are used to make the random variable η_{kl}.. The correlation coefficient $R(\eta_{ij}, \eta_{kl})$ of η_{ij} and η_{kl} is obtained by the computing process as follows:

$$R(\eta_{ij}, \eta_{kl}) = \frac{E\{(\eta_{ij} - E\{\eta_{ij}\})(\eta_{kl} - E\{\eta_{kl}\})\}}{\sqrt{D\{\eta_{ij}\}D\{\eta_{kl}\}}} = \frac{E\{(\eta_{ij} - \mu)(\eta_{kl} - \mu)\}}{\sigma^2} = \frac{1}{2\sigma^2}E\{[(\xi_i - \mu) + (\xi_j - \mu)][(\xi_k - \mu) + (\xi_l - \mu)]\}.$$

If random variables $\xi_i, \xi_j, \xi_k, \xi_l$ are different from each other, then variables $\xi_i, \xi_j, \xi_k, \xi_l$ are independent and hence $R(\eta_{ij}, \eta_{kl}) = 0$. If two of random variables $\xi_i, \xi_j, \xi_k, \xi_l$ are the same variable (assuming $\xi_i = \xi_k$), then the last term of above formula is simplified into $\frac{1}{2\sigma^2}D\{\xi_i\}$. Hence, $R(\eta_{ij}, \eta_{kl}) = 0.5$ holds.

For any two different random variables, η_{ij} and $\xi_k, i, j, k \in \{1,2,\cdots,n\}$, the correlation coefficient $R(\eta_{ij}, \xi_k)$ of η_{ij} and ξ_k is obtained by the computing process as follows:

$$R(\eta_{ij}, \xi_k) = \frac{E\{(\eta_{ij} - E\{\eta_{ij}\})(\xi_k - E\{\xi_k\})\}}{\sqrt{D\{\eta_{ij}\}D\{\xi_k\}}} = \frac{E\{(\eta_{ij} - \mu)(\xi_k - \mu)\}}{\sigma^2} = \frac{1}{\sqrt{2}\sigma^2}E\{[(\xi_i - \mu) + (\xi_j - \mu)](\xi_k - \mu)\}.$$

If random variables ξ_i, ξ_j, ξ_k are different from each other, then variables ξ_i, ξ_j, ξ_k are independent and hence $R(\eta_{ij}, \xi_k) = 0$. If two of random variables ξ_i, ξ_j, ξ_k are the same variable (assuming $\xi_i = \xi_k$), then the last term of above formula is simplified into $\frac{1}{\sqrt{2}\sigma^2}D\{\xi_i\}$. Hence, $R(\eta_{ij}, \xi_k) = \frac{\sqrt{2}}{2}$ holds. The theorem is proved.

From a size-n sample of a population, the data extending theorem illustrates that one can obtain a new random variable set whose size is $\frac{n(n-1)}{2}$. Each new random variable is of the same mean and variance with the population. This extending approach is very important in practical situations where many data points are needed (see Supplementary Table 8). In particular, the new

**Sample size**	12	14	16	18	20	22	24	26	28	30	32	34	36	38	40
**Extending set size**	66	91	120	153	190	231	276	325	378	435	496	561	630	703	780

Supplementary Table 8. Size of a simple V.S. size of the new random variable set. The extending set size is computed from the sample size n by the formula $\frac{n(n-1)}{2}$. In practical situations, given a small sample with its size 20, 190 new data points can be obtained. Increasing the size of a sample will quadratically increase the number of new data points.

random variables keep the same normal probability distribution for a normal distribution population that is often encountered in practical situations. Since new random variables are not independent, they can not be a new sample of the population. Although there exists certain correlation among new random variables and between the sample and new random variables, our empirical evidences and simulated examples indicate that good application effectiveness can be obtained from new random variables observations.

Particular details on implementing HMPR

If one independently repeated experimental observations N-times, the N input vectors $(x_{i1}, x_{i2}, \cdots, x_{im})$ and N response values y_i $(i = 1, 2, \cdots, N)$ of the system can be obtained. Treating the terms $x_{l_1}x_{l_2}\cdots x_{l_j}$ $(j = 1, 2, 3, \cdots)$ as new independent variables and using the N pairs of observations ($(x_{i1}, x_{i2}, \cdots, x_{im})$ and y_i), the pth-order multivariable polynomial function $HMP_p(x_1, x_2, \cdots, x_m)$ can be written into its vector form as follows:

$$Y = \Phi\Theta + \varepsilon,$$

where the response vector $Y = (y_1, y_2, \cdots, y_N)^T$, the parameter vector $\Theta = (\beta_0, \beta_1, \cdots, \beta_m, \cdots, \beta_{11\cdots 11}, \beta_{11\cdots 12}, \cdots, \beta_{mm\cdots m})^T$, and the error vector $\varepsilon = (\varepsilon_1, \varepsilon_2, \cdots, \varepsilon_N)^T$ are easy to be understood, and the complex loading matrix Φ is expressed as follows:

$$\Phi = \begin{bmatrix} 1 & x_{11} & \cdots & x_{1m} & \cdots & x_{11}^p & x_{11}^{p-1}x_{12} & \cdots & x_{1m}^p \\ 1 & x_{21} & \cdots & x_{2m} & \cdots & x_{21}^p & x_{21}^{p-1}x_{22} & \cdots & x_{2m}^p \\ \vdots & \vdots & \vdots & \vdots & \vdots & \vdots & \vdots & \vdots & \vdots \\ 1 & x_{N1} & \cdots & x_{Nm} & \cdots & x_{N1}^p & x_{N1}^{p-1}x_{N2} & \cdots & x_{Nm}^p \end{bmatrix}.$$

If the matrix $\Phi^T\Phi$ is invertible, the ordinary least square estimator $\Theta^* = (\Phi^T\Phi)^{-1}\Phi^T Y$ is the most frequent one to estimate polynomial coefficients. If the matrix $\Phi^T\Phi$ is not invertible, its singular value decomposition (SVD) and the truncated Least-Squares estimation (tLS) also can solve the parameter estimation problem. After substituting the estimated parameter vector Θ^* into the function $HMP_p(x_1, x_2, \cdots, x_m)$, the obtained p^{th}-order multivariable polynomial function $HMP_p^*(x_1, x_2, \cdots, x_m)$ can be used to predict the response variable y at a new input vector $(x_1^{new}, x_2^{new}, \cdots, x_m^{new})$. The 1th-order multivariable polynomial regression is the ordinary multivariable linear regression (MLR). It is the most striking difference between the HMPR and MLR that the HMPR nicely describes the complex nonlinearities of a nonlinear system by introducing nonlinear terms $x_{l_1}x_{l_2}\cdots x_{l_j}$ $(j = 1, 2, 3, \cdots)$. Deduced from the HMPR, a HMPM is the accurate computational system model that represents the analytical relationships between its response variable and input variables. This analytical relationships can visually tell people internal interactions of input variables and that which input variable has main contribution to the response variable. These simple and visual informations may provide scientists certain reference values to uncover mechanisms behind complex phenomena. All of those are exactly the reasons why we introduce the HMPR as an important methodological supplement to emotional estimation methodology.
